# The utility of ultra-widefield fluorescein angiography in pediatric retinal diseases

**DOI:** 10.1186/s40942-018-0122-2

**Published:** 2018-06-05

**Authors:** Charles M. Calvo, Mary Elizabeth Hartnett

**Affiliations:** John A. Moran Eye Center, University of Utah, 65 Mario Capecchi Dr., Salt Lake City, UT 84132 USA

**Keywords:** Ultra-widefield fluorescein angiography, Ultra-wide field imaging, Pediatric retina, Coats disease, Familial exudative vitreoretinopathy, Retinopathy of prematurity, Incontentia pigmenti

## Abstract

**Background:**

Ultra-widefield angiography is the latest technology in the evolution of fundus fluorescein angiography. With the ability to capture up to 200° of the fundus in a single image, far peripheral retinal pathology can be imaged. Generally, obtaining high-quality fundus fluorescein angiography in a child without sedation in the outpatient setting is exceedingly challenging. Therefore, there are advantages to imaging platforms that can capture the peripheral retina in young children without anesthesia. Often pediatric retinal diseases have pathology localized to the far periphery, which further validates the utility of ultra-widefield angiography. Ultra-widefield angiography has been successfully used without sedation for evaluation of children with various pediatric retinal diseases such as Coats disease, familial exudative vitreoretinopathy, and retinopathy of prematurity.

**Conclusion:**

This non-contact, non-mydriatic modality has been utilized in the evaluation of pediatric retinal diseases and demonstrated to have benefits over conventional fluorescein angiography techniques.

## Background

For nearly six decades, fluorescein angiography has been a diagnostic staple in the diagnosis and management of vitreoretinal diseases, graphically demonstrating information regarding circulations of the retina, choroid and optic nerve head, and barrier integrity of the neurosensory retina and retina pigment epithelium. Capturing quality angiographic images of the peripheral retina with a 30°–60° field of view of most standard fundus cameras proved difficult [1]. This requires a skilled photographer and a cooperative patient able to maintain steady gaze in various directions. This challenge is particularly true when imaging pediatric patients. Retinal diseases affecting the pediatric population include a number of genetic and environmentally influenced conditions that involve the peripheral retina, including as examples, retinopathy of prematurity (ROP), familial exudative vitreoretinopathy (FEVR) and Coats disease [2–4]. Information regarding the perfusion of the retina, vascular leakage, and neovascularization obtained by fluorescein angiography are paramount to the management of proliferative and exudative pediatric retinal diseases. Therefore, there has been an evolution of imaging modalities to better capture the peripheral retina with fluorescein angiography.

Techniques were developed to create montages of images from several fields captured by fundus photography to widen the field of view capable with standard 30 or 50 degree cameras. The 7 Standard Fields (7SF) protocol for fundus photography was developed and popularized by the Diabetic Retinopathy Study group expanded visualization to approximately 75° with 3 photos horizontally across the macula and 4 photos surrounding the optic disc [5]. Similarly, a 9 Standard Fields protocol was used in monitoring cytomegalovirus retinitis [6]. However, the time sensitive nature of fluorescein angiography in addition to the technical difficulty capturing images by photography to create montages made such a technique cumbersome and still often left large areas of peripheral retina not visualized. This technique was very difficult in children or infants.

Widefield angiography, capturing > 30° to < 200° [7], was developed as a solution to the limitations of standard angiography with traditional fundus cameras. The Staurenghi contact lens system (Ocular Staurenghi 230 SLO Retina Lens; Ocular Instruments Inc, Bellevue, WA, USA) achieved imaging up to 150° with a standard confocal scanning laser ophthalmoscope [8]. The contact lens requires patient cooperation because of the direct corneal contact which is a challenge for adult patients but particularly difficulty for most pediatric patients. The Pomerantzeff Equator-Plus camera [9] and the Panoret) CMT Medical Technologies Inc, Valley Stream, NY( [10] visualized 148° and 130° of the retina, respectively; however, their popularity was limited by the need for mydriasis, contact lenses, and that both systems are stand-alone instruments that cannot be integrated with existing fundus cameras. The Retcam (Clarity Medical Systems, Pleasanton, California, USA) portable contact-based camera, now in its third generation, with a 130° field of view entered the market in 1997 and made its niche in pediatric ophthalmology and pediatric retina [11]. The portability of the Retcam allowed for bedside and operating room imaging of pediatric patients and the handheld contact camera allowed for manual rotation of globe in very young patients or those under anesthesia. Infants can undergo imaging with Retcam in the office with swaddling, lid speculum, and topical anesthesia but older children most commonly require general anesthesia.

The Optos camera (Optos 200Tx, Dunfermline, Scotland, UK) was the first ultra-widefield imaging system, which produces a 200° view of the retina (about 82% of the surface area) [12, 13]. In addition, the Optomap auto-montage software can image 220° or about 97% of the retina. A confocal scanning laser ophthalmoscope is combined with an ellipsoidal mirror to image the retinal periphery with one capture without the need for a contact lens, and most often, without mydriasis. Some of the limitations of the Optos platform are the distortion and decreased resolution of the far periphery as well as reduced superior and inferior fundus imaging in comparison to the temporal and nasal fundus [14]. More recently, Heidelberg (Heidelberg Engineering, Germany) released their noncontact lens system that attaches to the Heidelberg Spectralis and Retina Angiograph platforms to achieve ultra-widefield photography and angiography [15].

## Pediatric conditions imaged with ultra-widefield angiography

As discussed above, in the pediatric population, imaging of the peripheral retinal often required general anesthesia. Initially, standard 30° or 45° cameras were used and required positioning of the eye to the periphery in children who were unable to cooperate. Contact cameras were helpful but also required general anesthesia. However, with UWFA, there is the ability to obtain quality images in some children without the need for general anesthesia. We describe several conditions below in which UWFA has provided adequate images for diagnosis and treatment without the need for general anesthesia. The PubMed database was systematically searched using the search terms [(pediatric or pediatric retina or Coats or familiar exudative retinopathy) and (fluorescein angiography or widefield imaging or ultra-widefield imaging)]. To our knowledge, all articles or abstracts describing the use of ultra-widefield fluorescein angiography in the pediatric population were included in this review (Table 1).

**Table 1:** Summary of UWFA reports

Author	# of patients	Patient ages	Diagnoses	Notable conclusions
Kang et al.	8	9, 10, 10, 11, 11, 14, 14, 14	FEVR, Coats	A significant degree of pathology was outside of 7 Standard Fields overlay
Tsui et al.	16	5, 5, 6, 6, 7, 8, 8, 9, 10, 11, 11, 12, 12, 12, 12, 12	Coats, pars planitis X-linked retinoschisis, retinal dystrophy, choroidal melanoma, juvenile idiopathic arthritis, toxoplasmosis, panuveitis	Eyelash artifact was present in 12 of 40 images (30%)
Rabiolo et al.	5	8, 8, 12, 13, 15	Coats	77.8% of unaffected eyes had peripheral abnormalities such as nonperfusion and telangiectasia
Patel et al.	1	3 months old	Incontentia pigmenti	‘Flying baby’ technique was used to position the child. Oral 2% fluorescein solution was safely used
Fung et al.	3	Infant born at 27 weeks	ROP	‘Flying baby’ technique used; NICU team present to monitor child in this position
		Infant born at 24 weeks		
		Infant born at 29 weeks		

### Familial exudative vitreoretinopathy

**Fig. 1:**
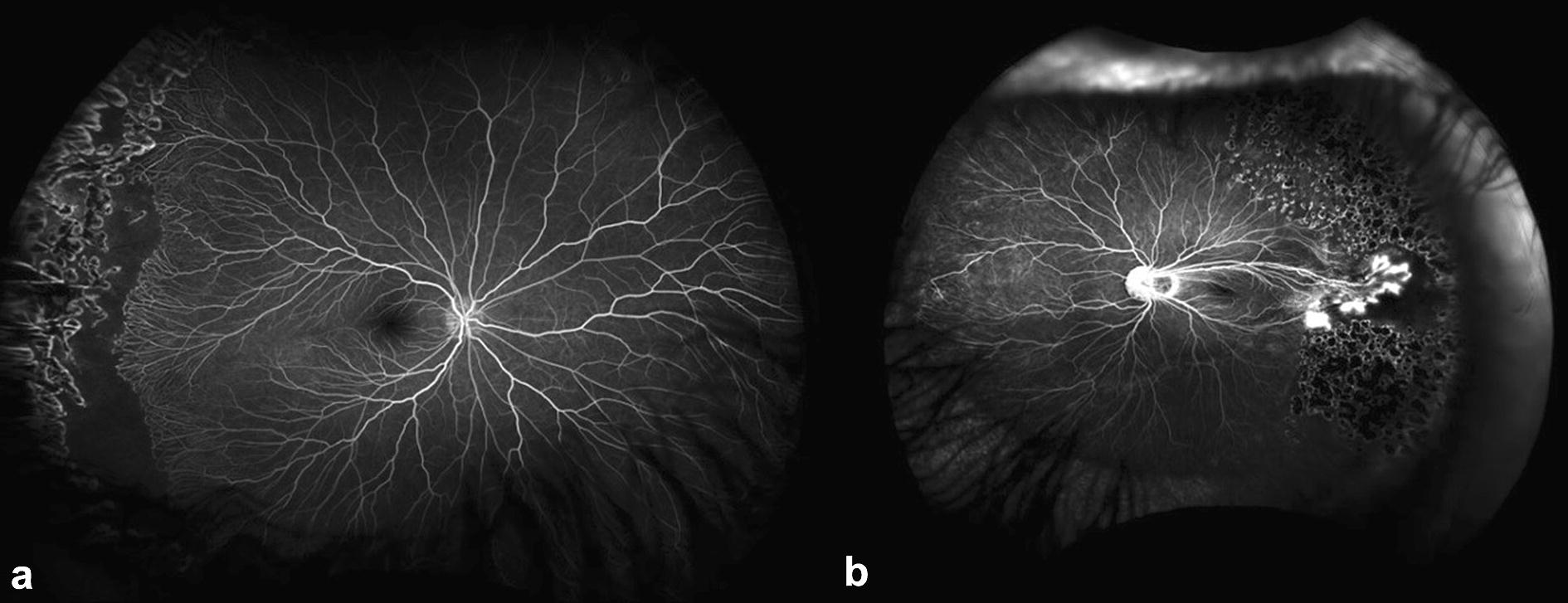
Representative photo of ultra-widefield angiography of two children with familial exudative vitreoretinopathy. **a** Right eye of a 9 year old child with persistent retinal nonperfusion posterior to prior laser photocoagulation. **b** Left eye of a 10 year old child with prominent temporal vascular dragging, hyperfluorescence and leakage from neovascularization

Familial exudative vitreoretinopathy (FEVR) has a variable presentation, including straightened retinal vessels, temporal dragging of the macula, abnormal retinal vascular permeability (Fig. 1), described as late-phase angiographic posterior and peripheral vascular leakage (LAPPEL), and capillary/vascular non-perfusion, which may be a consequence of leaky retinal vessels. Capillary nonperfusion can progress toward the macula and threaten vision or be associated with later intravitreal neovascularization, which can lead to complex tractional retinal detachments. Both increased permeability and vitreous traction from intravitreal neovascularization can lead to vision loss and blindness [16].

In FEVR, myopia is present and family history should be sought as genetic mutations are recognized in about 50% of cases, but the condition is variable in expressivity so even a normal parent may pass onto offspring a mutation manifesting as disease. Many of the early findings of FEVR occur in the far peripheral retina making the diagnosis challenging. The findings are best appreciated with fluorescein angiography that demonstrates capillary non-perfusion and leakage of the vascular front in the region between vascularized and avascular retina. There is also intravitreal neovascularization in Stage 2 FEVR (Table 2) that shows exuberant leakage in late stages. UWFA permits good visualization of the nasal and temporal peripheral retinas even in some children. In a series of five patients ranging from 11 to 14 years of age, ultra-widefield fundus photography and ultra-widefield fluorescein angiography were used to successfully capture angiographic images in all [17]. Laser photocoagulation was performed in 2 eyes of 2 patients with UWFA guiding treatment to target areas of peripheral non-perfusion. Repeated UWFA allowed for identification of regions of untreated non-perfusion and guided the second treatment session of laser photocoagulation. Kang et al. concluded that standard 7SF fluorescein angiography would not have identified these areas of pathology. There were no reported complications or loss of vision.

**Table 2:** Revised familial exudative vitreoretinopathy clinical staging system [37]

Stage	Description
1	Avascular periphery or anomalous intraretinal vascularization
1a	Without exudate or leakage
1b	With exudate or leakage
2	Avascular retinal periphery with extraretinal vascularization
2a	Without exudate or leakage
2b	With exudate or leakage
3	Extramacular retinal detachment
3a	Without exudate or leakage
3b	With exudate or leakage
4	Macula-involving retinal detachment
4a	Without exudate or leakage
4b	With exudate or leakage
5	Total retinal detachment
5a	Open funnel
5b	Closed funnel

### Coats disease

**Fig. 2:**
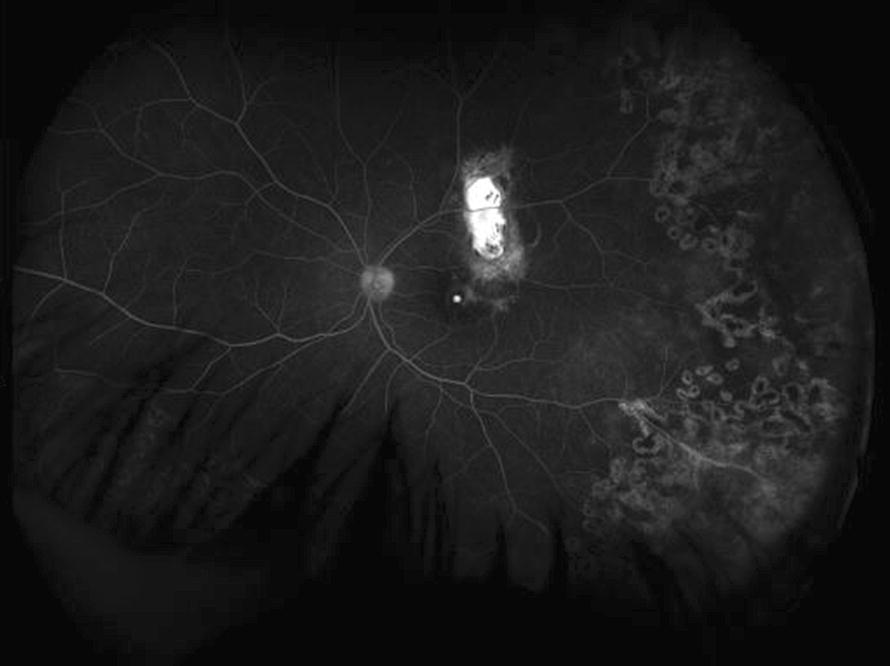
Representative photo of ultra-widefield angiography in an 8 year old child with Coats disease. Laser photocoagulation was performed previously to telangiectatic vessels and there is staining of chronic exudation in the superior macula

Coats disease presents with symptoms in one eye usually and is more common in boys than in girls (~ 80 vs. 20%). Contact Retcam imaging has identified peripheral areas of nonperfused retina in the fellow eye of patients with Coats disease, which is more than three standard deviations greater than the < 1 disc diameter of non-pathologic, nonperfused, temporal retina in normal, young children [18]. Other vascular abnormalities such as telangiectasias and microaneurysms can be found in the fellow eyes of patients with Coats but these are often asymptomatic [18]. When bilateral symptomatic Coats disease does occur, it can be in association with fascioscapular muscular dystrophy. Treatment of Coats disease is often with laser directly to abnormal telangiectatic vasculature and scatter treatment to the attached nonperfused retinal areas in early stages of Coats disease (Fig. 2; Table 3). In stages 3 and 4 Coats disease, external drainage of subretinal fluid sometimes in combination with vitrectomy and silicone oil are used.

**Table 3:** Coats disease staging classification [38]

Stage	Description
1	Retinal telangiectasia only
2	Telangiectasia and exudates
2a	Extrafoveal exudation
2b	Foveal exudation
3	Exudative retinal detachment
3a	Subtotal retinal detachment (1) extrafoveal (2) foveal
3b	Total retinal detachment
4	Total retinal detachment and glaucoma
5	Advanced end-stage disease

UWFA has been extensively utilized in the evaluation and management of Coats disease [17, 19, 20]. Three children with the diagnosis of Coats disease ranging from 9 to 14 years of age underwent UWFA. In an overlay of 7 Standard Fields protocol to UWFA images, all areas of retinal telangiectasia and non-perfusion were outside of the borders of the 7 Standard Fields. UWFA-guided laser photocoagulation resulted in reduction in exudation, resolution of leaking telangiectatic vessels, and decreased macular edema demonstrated by repeat UWFA. Tsui et al. demonstrated the outpatient use of UWFA without sedation in younger children, aged 6 and 7 years old, with Coats disease [19]. Targeted laser photocoagulation was also performed. In a report by Rabiolo et al., five children and teenagers (ages 8–15) received UWFA imaging in an outpatient setting [20]. Only 1 child was treatment naïve with all other patients having been previously treated with laser photocoagulation or cryotherapy. In addition to concluding that UWFA captured more peripheral retinal pathology than standard FA, the authors determined by UWFA that 77.8% of asymptomatic fellow eyes had far peripheral nonperfusion and capillary telangiectasias.

## Incontentia pigmenti

Incontinentia pigmenti is an inherited X-linked dominant disease, mainly seen in females as it is lethal in males. Avascular retina is seen and vessels can develop and remodel normally but if intravitreal neovascularization occurs, there is a risk of vision loss from vitreous hemorrhage and tractional retinal detachment. Vision loss also occurs from avascularity in the macula [21]. Laser treatment can cause regression of neovascularization and reduce the risk of later retinal detachment. Fluorescein angiography is useful to diagnose non-perfused retina and leakage of fluorescein from intravitreal neovascularizaiton, but the condition manifests in infancy often, and imaging requires general anesthesia for early diagnosis and management. There is a single report demonstrating the application of UWFA for the evaluation of incontentia pigmenti [22]. A 3 month old infant girl underwent UWFA in an outpatient office setting. Oral fluorescein 2% solution was given at a dose of 25 mg/kg via a bottle with a mixture of infant formula milk 30 min prior. No sedation was provided. Following standard pupillary dilation and placement of a lid speculum, the child was held in a ‘flying baby’ position with the body and head supported and the technician aligning the infant’s eye with the lens. In under 5 min, successful angiography was performed with images of the far periphery in all quadrants. Retinal ischemia, arteriovenous shunting, and neovascularization were well documented. There were no reported complications from the oral fluorescein or the positioning of the child.

## Retinopathy of prematurity

Retinopathy of prematurity (ROP) is a leading cause of blindness and vision loss worldwide in premature infants. ROP is identified by screening infants at risk and monitoring until physiologic vascularization has extended to the ora serrata. Type I ROP is treated with laser photocoagulation or intravitreal anti-VEGF agents. In the past, fluorescein angiography was not standard care for ROP [23, 24]. However, with the advent of agents that inhibit the bioactivity of vascular endothelial growth factor (VEGF) and studies that test efficacy and safety of dose, it has been realized that anti-VEGF agents can change the natural history of ROP and that intravitreal neovascularization and later tractional retinal detachments occur even a year after an injection [25]. Fluorescein angiography has increasingly been used to understand the pathophysiologic course of ROP following intravitreal anti-VEGF agents [26–28]. In the only report of the use of UWFA in infants with retinopathy of prematurity (ROP), Fung and colleagues published a consecutive case series of 3 infants with ROP born 24–29 weeks gestation that underwent UWFA in an outpatient office setting [29]. UWFA was performed with the ‘flying baby’ technique supporting the head and baby so the eye could be imaged following administration of oral fluorescein and without any sedation. Of note, a neonatal intensive care team was present for monitoring of the child during the procedure. High-resolution angiograms were obtained in 3 of 3 infants and no complications were reported, however, the authors noted the prone, ‘flying baby’ could result in cardiopulmonary complications in these medically-fragile infants. The authors concluded that UWFA allowed for imaging significantly more of the peripheral retina in a single image than the 130° fundus imaging of the Retcam camera.

## Miscellaneous

UWFA has also been successfully used in the evaluation of children with X-linked retinoschisis, Stargardt disease, Best’s disease, toxoplasmosis chorioretinitis, juvenile sarcoidosis, choroidal melanoma, juvenile idiopathic arthritis, pars planitis, and traumatic retinal detachment [19]. Ages ranged from 5 to 12 years old.

## Conclusions

Ultra-widefield fluorescein angiography has established itself as a useful tool to the vitreoretinal specialist and is proving to be valuable in the care of pediatric retina patients. There is mounting evidence that UWFA may be a valuable adjunct to identify peripheral retinal pathology undetected by the conventional 75° field from 7 Standard Fields in the evaluation of pediatric retinal diseases [17]. There is a clear benefit in avoiding examinations under anesthesia for those children who may be able to cooperate with noncontact angiography. Repeated general anesthesia may pose a significant cardiopulmonary risk to systemically ill children. There have been concerns raised about the effect of repeated general anesthesia on cognitive development [30–32]. Additionally, in-office UWFA is likely to be less stressful for the child’s family and less time-consuming for all parties involved.

However, there are definite limitations of UWFA. Poor image quality, distortion, and artifact may affect up to 5–10% of images in some studies [33, 34]. Naturally, this distortion is related to the two-dimensional representation of the rounded, three-dimensional surface of the fundus. However, a method for rectifying peripheral distortions when calculating retinal area and measuring lesions has been described [35]. Inferior eyelash artifact was present in 12 of 40 images (30%) in one study [19]. The Optos platform does not image the far superior and inferior peripheral retina as extensively as compared with its imaging of the temporal and nasal retina [14]. The Heidelberg Spectralis was shown to image the superior and inferior retinal vasculature more peripherally than the Optos Optomap in nine of ten eyes (18 of 20 quadrants); however, the Optos Optomap could image the nasal and temporal retinal vasculature more peripherally in ten of ten eyes (20 of 20 quadrants) [15]. Though demonstrated to be used successfully in three infants with ROP and one infant with incontinentia pigmenti, it may not be safe to routinely image such children in the office with the required precarious positioning.

Ultra-widefield angiography is a valuable adjunct to traditional forms of fluorescein angiography as the technique proves to be less invasive and better tolerated in pediatric patients. The greater ability of UWFA to identify peripheral retinal pathology over conventional angiography platforms is especially suited to pediatric retinal conditions such as Coats disease and FEVR. The future will hold better and less invasive methods of imaging the peripheral retina vasculature such as ultra-widefield optical coherence tomography (OCT) angiography [36]. Further studies utilizing UWFA are warranted to integrate UWFA into management recommendations for pediatric retinal conditions.

## Abbreviations

UWFA: ultra-widefield fluorescein angiography
ROP: retinopathy of prematurity
FEVR: familial exudative vitreoretinopathy
7SF: 7 Standard Fields
